# Inhibition of Orai Channel Function Regulates Mas-Related G Protein-Coupled Receptor-Mediated Responses in Mast Cells

**DOI:** 10.3389/fimmu.2021.803335

**Published:** 2022-01-20

**Authors:** Shaswati Chaki, Ibrahim Alkanfari, Saptarshi Roy, Aetas Amponnawarat, Yvonne Hui, Carole A. Oskeritzian, Hydar Ali

**Affiliations:** ^1^ Department of Basic and Translational Sciences, University of Pennsylvania School of Dental Medicine, Philadelphia, PA, United States; ^2^ Faculty of Dentistry, King AbdulAziz University, Jeddah, Saudi Arabia; ^3^ Department of Family and Community Dentistry, Faculty of Dentistry, Chiang Mai University, Chiang Mai, Thailand; ^4^ Department of Pathology, Microbiology and Immunology, University of South Carolina School of Medicine, Columbia, SC, United States

**Keywords:** Calcium release-activated calcium (CRAC), Orai, Mast cells, Mas-related G protein-coupled receptor X2 (MRGPRX2), Mas-related G protein-coupled receptor B2 (MrgprB2), Substance P

## Abstract

Mast cells (MCs) are tissue resident immune cells that play important roles in the pathogenesis of allergic disorders. These responses are mediated *via* the cross-linking of cell surface high affinity IgE receptor (FcϵRI) by antigen resulting in calcium (Ca^2+^) mobilization, followed by degranulation and release of proinflammatory mediators. In addition to FcϵRI, cutaneous MCs express Mas-related G protein-coupled receptor X2 (MRGPRX2; mouse ortholog MrgprB2). Activation of MRGPRX2/B2 by the neuropeptide substance P (SP) is implicated in neurogenic inflammation, chronic urticaria, mastocytosis and atopic dermatitis. Although Ca^2+^ entry is required for MRGPRX2/B2-mediated MC responses, the possibility that calcium release-activated calcium (CRAC/Orai) channels participate in these responses has not been tested. Lentiviral shRNA-mediated silencing of Orai1, Orai2 or Orai3 in a human MC line (LAD2 cells) resulted in partial inhibition of SP-induced Ca^2+^ mobilization, degranulation and cytokine/chemokine generation (TNF-α, IL-8, and CCL-3). Synta66, which blocks homo and hetero-dimerization of Orai channels, caused a more robust inhibition of SP-induced responses than knockdown of individual Orai channels. Synta66 also blocked SP-induced extracellular signal-regulated kinase 1/2 (ERK1/2) and Akt phosphorylation and abrogated cytokine/chemokine production. It also inhibited SP-induced Ca^2+^ mobilization and degranulation in primary human skin MCs and mouse peritoneal MCs. Furthermore, Synta66 attenuated both SP-induced cutaneous vascular permeability and leukocyte recruitment in mouse peritoneum. These findings demonstrate that Orai channels contribute to MRGPRX2/B2-mediated MC activation and suggest that their inhibition could provide a novel approach for the modulation of SP-induced MC/MRGPRX2-mediated disorders.

## Introduction

Mast cells (MCs) are tissue resident immune cells best known for their roles in anaphylaxis and atopic disorders, which result from FcϵRI/IgE-mediated histamine release and the generation of lipid mediators and cytokines (1). It is well documented that the release of calcium (Ca^2+^) from intracellular stores and its subsequent influx through store-operated calcium (SOC) channels are required for IgE-mediated release of newly synthesized mediators (2). Human and murine MCs express the endoplasmic reticulum (ER)-resident protein stromal interaction molecule-1 and -2 (STIM-1/2) that sense the depletion of intracellular Ca^2+^, resulting in the activation of calcium release-activated calcium (CRAC) channels such as CRACM1, CRACM2 and CRACM3 (also known as Orai1, Orai2 and Orai3) (3–6). Utilization of gene silencing and pharmacological approaches has demonstrated that Orai channels contribute to IgE-mediated degranulation in human lung MCs *in vitro* and bronchoconstriction *ex vivo* (6–8).

While all MCs are characterized by the expression of cell surface FcϵRI, cutaneous MCs highly expresses a newly identified G protein-coupled receptor (GPCR) known as Mas-related GPCR-X2 (MRGPRX2, mouse counterpart MrgprB2) (9). Activation of this receptor by an increasing list of cationic ligands contributes to host defense, pseudoallergy and a number of chronic inflammatory diseases (9–11). Substance P (SP)-mediated activation of human skin MCs *via* MRGPRX2 is implicated in the pathogenesis of chronic urticaria and mastocytosis (12, 13). Moreover, activation of murine MCs by SP *via* MrgprB2 contributes to experimental neurogenic inflammation, pain, and atopic dermatitis (14, 15). Although both anti-IgE and SP cause intracellular increase in Ca^2+^ concentration, the kinetics of Ca^2+^ mobilization varies for different stimuli. Stimulation of human peripheral blood-derived cultured MCs (PBCMCs) with anti-IgE causes more sustained Ca^2+^ mobilization compared to SP. This is correlated with IgE-dependent progressive degranulation with larger granule size while SP stimulation results in faster degranulation with smaller granule size (16). However, the mechanism *via* which SP activates MRGPRX2 to cause Ca^2+^ mobilization and mediator release is unknown.

The purpose of this study was to determine if Orai channels contribute to SP-induced signaling and mediator release in human and murine MCs *in vitro* and MrgprB2-mediated inflammation *in vivo*. We utilized complementary approaches, namely, shRNA-mediated knockdown of Orai1, Orai2 and Orai3 in human MCs line, LAD2 and an Orai inhibitor, Synta66, in human skin-derived MCs and mouse peritoneal MCs for *in vitro* studies. We also tested the effects of Synta66 on SP-induced cutaneous vascular permeability and leukocyte recruitment in mouse peritoneum. This study provides novel insights on the role of Orai channels on MC activation by SP with potential implications for modulating MRGPRX2-mediated disorders.

## Materials and Methods

### Reagents

All cell culture reagents were purchased from Invitrogen (Carlsbad, CA, USA); recombinant human stem cell factor (rhSCF), mouse interleukin-3 (mIL-3), and mouse stem cell factor (mSCF) were from PeproTech (Rocky Hill, NJ, USA); p-nitrophenyl-N-acetyl-β-D-glucosamine (PNAG) was from Sigma-Aldrich (St. Louis, MO, USA) and Fura-2 acetoxymethyl ester was from Abcam (Cambridge, MA, USA). Substance P (SP) was from AnaSpec (Fremont, CA, USA). Phycoerythrin-conjugated anti-MRGPRX2, FITC-conjugated anti LAMP-1 and all other flow cytometry antibodies were from Biolegends (San Diego, CA, USA). Rabbit anti-Orai1, Orai2 and Orai3 antibodies from Alomone lab (Rockville, MD, USA), anti-ERK1/2, anti-phospho-ERK1/2 (Thr-202/Tyr-204), anti-phospho-Akt (Ser-473), anti-Akt, β-Actin and goat anti-rabbit IgG-HRP were obtained from Cell Signaling Technology (Danvers, MA, USA). SuperSignal West Pico Maximum Sensitivity Substrate was from Thermo Scientific (Rockford, IL, USA). Synta66 (3-fluoro-pyridine-4-carboxylic acid (2,5-dimethoxy-biphenyl-4-yl)-amide) was purchased from Calbiochem (San Diego, CA, USA). ELISA kits for mouse TNF-α, and human TNF-α, IL-8, CCL-3 were obtained from R&D system (Minneapolis, MN, USA). BCA Protein Assay Kit was obtained from Pierce Biotechnology (Rockford, IL, USA).

### Mice

C57BL/6 (WT) mice were obtained from the Jackson Laboratory (Bar Harbor, ME, USA). MrgprB2^−/−^ mice were generated *via* CRISPR/Cas9 by CRISPR core of University of Pennsylvania (17). All mice were housed under specific pathogen-free conditions on autoclaved hardwood bedding. Both male and female mice (8–10 weeks old) were used for experiments. All experiments were approved by the Institutional Animal Care and Use Committee at University of Pennsylvania.

### Cell Line Cultures

The human MC line LAD2 was provided by Dr. A. Kirshenbaum and Dr. D. Metcalfe (Laboratory of Allergic Diseases, National Institute of Allergy and Infectious Diseases, National Institutes of Health, Bethesda, MD, USA) and was maintained in complete StemPro-34 medium supplemented with L-glutamine (2 mM), penicillin (100 IU/ml), streptomycin (100 μg/ml), and rhSCF (100 ng/ml). Hemidepletion was performed weekly with media containing rhSCF (18).

### Human Skin-Derived Primary Mast Cell Isolation and Culture

Human skin surgical sample was collected from the Cooperative Human Tissue Network of the National Cancer Institute, as approved by the Internal Review Board at the University of South Carolina. Skin MCs were harvested and cultured as previously described (18, 19). Briefly, subcutaneous fat was removed by blunt dissection, and residual tissue was cut into 1 to 2 mm fragments and digested with type 2 collagenase (1.5 mg/ml), hyaluronidase (0.7 mg/ml), and DNase I (0.3 mg/ml) in Hank’s Balanced Salt Solution (HBSS) for 2 h at 37°C. The dispersed cells were collected by filtering through a 70-µm cell strainer and resuspended in HBSS containing 1% fetal calf serum (FCS) and 10 mM HEPES to stop the reaction. Cells were resuspended in HBSS and layered over 75% Percoll in a HBSS cushion and centrifuged at 800×*g* at room temperature for 20 min. Nucleated cells were collected from the buffer/Percoll interface. Percoll gradient-enriched cells were resuspended at a concentration of 1 × 10^6^ cells/ml in serum-free X-VIVO 15 medium containing 100 ng/ml rhSCF. MCs were used after 6–10 weeks of culture, when purity was nearly 100%, as confirmed with toluidine blue staining.

### Isolation of Mouse Peritoneal MCs

Mouse peritoneal cells were isolated by intraperitoneal lavage from 8 to 10 weeks old C57BL/6 and MrgprB2^-/-^ mice weighing ~20 g as described previously (20). Briefly, the peritoneal cavity was lavaged with 10 ml sterile cold HBSS supplemented with 3% FCS and 10 mM HEPES, pH 7.2. The cells were cultured in Roswell Park Memorial Institute (RPMI 1640) medium supplemented with 10% FCS, murine IL-3 (10 ng/ml), and murine SCF (30 ng/ml). After 48 h, non-adherent cells were removed, and adherent cells were cultured in fresh medium for an additional 4–6 weeks. Suspension cells were used for experiments as peritoneal MCs (PMCs).

### Generation and Purification of Scramble shRNA, Orai1 shRNA, Orai2 shRNA and Orai3 shRNA and Knockdown in LAD2 Cells Using Lentiviral Transduction

To inhibit Orai1, Orai2, and Orai3 protein expression in human MCs, lentiviral shRNA-mediated knockdown was performed in LAD2 cells. Orai1, Orai2, and Orai3 targeted Mission shRNA lentiviral plasmids were purchased from Sigma-Aldrich. The clone that gave highest knockdown efficacy for Orai1 (TRCN0000413611), Orai2 (TRCN0000166201), and Orai3 (TRCN0000165405) were used for the study. A nontargeted scramble vector (SHC002) was used as a control. Nontargeted control or respective Orai knockdown plasmids were packaged into virus particle using HEK293T cells. Targeted shRNA plasmid (4.5 µg) along with helper plasmid pCMV-VSV-G (Addgene# 8454, 0.57 µg) and pCMV-dR8.2 (Addgene# 8455, 4.5 µg) were transfected in HEK293T cells (6 × 10^6^ cells/transfection) in T75 flask. After 72 h, viral particles were harvested, filtered through 0.45 µm filter and concentrated using Lenti-X™ concentrator (Takara Bio Inc, Japan). Cell transduction was performed by mixing 1.5 ml of concentrated virus particles with 3.5 ml of LAD2 (10 × 10^6^) cells. After 8 h incubation in 37°C and 5% CO_2_, medium was changed to complete StemPro34 media and antibiotic puromycin (2 µg/ml; Sigma) was added after 16 h (21). Western blotting was performed to confirm knockdown and experiments were performed 4 days after initiation of puromycin selection.

### Degranulation Measured by β-Hexosaminidase Release Assay

The degranulation was measured by β-hexosaminidase release as described previously (21). Briefly, LAD2 cells (1 × 10^4^), human skin-derived MCs (5 × 10^3^), and PMCs (1 × 10^4^) were seeded into a 96-well, white, clear-bottom cell culture plate in HEPES buffer containing 0.1% bovine serum albumin (BSA) and stimulated with differing concentrations of SP for 30 min at 37°C. Cells without treatment were designated as control. To determine the total β-hexosaminidase content, unstimulated cells were lysed in 50 μl of 0.1% Triton X-100. Aliquots (20 μl) of supernatants or cell lysates were incubated with 20 μl of 1 mM p-nitrophenyl-*N*-acetyl-β-*D*-glucosamine (PNAG) for 1.15 h at 37°C. The reaction was stopped by adding 250 μl of stop solution (0.1 M Na_2_CO_3_/0.1 M NaHCO_3_). For assays using CRAC inhibitor (Synta66), cells were incubated for 5 min with the suitable dose of inhibitor prior to agonist stimulations. The absorbance was measured with a microplate reader at a wavelength of 405 nm using Versamax microplate spectrophotometer (Molecular Devices, San Jose, CA, USA). Data was represented as percent degranulation by diving the β-hexosaminidase release in sample with total β-hexosaminidase release.

### Degranulation Measured by the Surface Expression of Lysosomal-Associated Membrane Protein 1 (LAMP-1)

Degranulation was also assessed by flow cytometric measurement of the surface expression of LAMP-1 (20). Murine PMCs (3 × 10^5^) were stimulated with SP (100 µM) for 10 min in HEPES buffer containing 0.1% BSA, washed and exposed to FITC-conjugated anti-LAMP-1 antibody in FACS buffer (PBS containing 2% FCS and 0.02% sodium azide) for 30 min on ice in dark. For experiments involving inhibitors, PMCs were incubated with Synta66 (10 µM) for 5 min prior to SP stimulation. Cell surface expression of LAMP-1 was measured by flow cytometry using a BD LSR II flow cytometer (San Jose, CA) and analyzed with the FlowJo software version 10.7.2 (Tree Star Inc., Ashland, OR). The adjusted mean fluorescent intensity (MFI) was calculated as a ratio of the MFI of sample to the MFI of isotype control.

### Calcium Mobilization

LAD2 (0.3 × 10^6^ cells) cells were loaded with 1 μM Fura-2 acetoxymethyl ester in Ca^2+^-free HEPES-buffered saline containing 0.1% BSA for 30 min in the dark at 37°C, followed by de-esterification for additional 15 min at room temperature. Cells were washed, resuspended in Ca^2+-^free buffer and Ca^2+^ mobilization was measured for 25 min with addition of SP at 100 s and reintroduction of Ca^2+^ at 400 s. For assay with inhibitor, cells were loaded with Fura-2 acetoxymethyl ester (Fura-2AM) and incubated with 10 µM of Synta66 for 30 min in Ca^2+-^free buffer prior to ligand stimulation. Calcium signals was determined using a Hitachi F-2700 Fluorescence Spectrophotometer with dual excitation wavelength of 340 and 380 nm, and an emission wavelength of 510 nm at every 2 s (22). For the assay with human skin MCs, cells (0.2 × 10^6^) were loaded with Fura-2 acetoxymethyl ester similarly in presence and absence of Synta66, and Ca^2+^ mobilization was measured in Varioskan LUX Multimode Microplate Reader (ThermoScientific, Waltham, MA, USA).

### Western Blotting

Western blotting was performed as described previously (17). Briefly, control and Orai shRNA transduced LAD2 cells (1 × 10^6^) were lysed in radioimmunoprecipitation assay buffer (1× RIPA) with protease inhibitor cocktail, and the protein concentration was measured by the BCA protein assay. Twenty five micrograms of protein samples were applied to 10% sodium dodecyl sulfate-polyacrylamide gel electrophoresis (SDS-PAGE) and subsequently transferred to PVDF membrane. After brief blocking (5% skim milk, 1 h), blots were incubated with antibodies against Orai1 (1:500), Orai2 (1:500), Orai3 (1:500) and β-Actin (1:1,000) at 4°C overnight. Blots were incubation with specific horseradish peroxidase (HRP)-conjugated secondary antibodies for another hour, followed by incubation with an HRP substrate for ECL and image captured on iBRIGHT 1500 (ThermoFisher, Waltham, MA, USA). Signal quantitation was carried out after normalization to β-Actin loading controls, as indicated. For the assay with Orai inhibitor, LAD2 cells (2 × 10^6^) were preincubated with Synta66 (10 µM) for 30 min, stimulated with SP (1 µM) for different time intervals (0, 5, 15 and 30 min) and cell lysates were prepared. Protein samples were run in SDS-PAGE, incubated with anti-phospho-pERK1/2 (1:2,000), anti-phospho-Akt (1:1,000), anti-ERK1/2 (1:2,000), and anti-Akt (1:1,000) antibodies and processed similarly.

### ELISA (Enzyme-Linked Immunosorbent Assay)

ELISAs were performed according to the manufacturer’s protocol (DuoSet ELISA kits, R&D systems) to quantify the release of murine TNF-α and human TNF-α, IL-8 and CCL-3. Briefly, LAD2 cells were washed once in serum-free Stem-Pro™-34 medium, suspended in complete medium and seeded in 24-well sterile plate (0.3 × 10^6^ cells/well) with appropriate concentration of agonist stimulation for 24 h. For experiments involving Synta66, cells were preincubated with inhibitor for 30 min prior to agonist stimulation. After 24 h, cells were centrifuged, and supernatants were collected to measure cytokines by ELISA. The expression of mTNF-α was measured in serum samples from mice untreated or treated with Synta66 and SP.

### Murine Evans Blue Dye Extravasation Model

Evans blue extravasation studies were performed on male and female C57BL/6 mice (8–10 weeks of age and weighing 20–25 g). Mice were anesthetized using Ketamine : Xylazine and intraperitoneally (i.p) injected with Synta66 (10 mg/kg in 50 µl DMSO) following intravenous (i.v.) injection with 0.1 ml of 1% Evans blue in saline. After 1 h, intradermal injection of SP (50 µM in 20 µl saline) was performed into the one ear, while vehicle was injected in other side. After 30 min, mice were euthanized; ear tissues were harvested, weighed, immersed in 500 µl of formamide and incubating overnight at 56°C. Each tissue solution was centrifuged at 10,000 rpm for 10 min; Evans blue dye extravasation was determined by collecting 200 µl of the supernatant and the OD values were measured at 650 nm using Versamax microplate spectrophotometer (20).

### *In Vivo* Murine Peritonitis Model

In short, 8–10 weeks old C57BL/6 mice (male and female) were intraperitoneally (i.p) injected with 50 µl of Synta66 (10 mg/kg in DMSO) or vehicle. After 1 h, peritonitis was induced by intraperitoneal injection of SP (50 µl of 200 µM SP in saline) or vehicle. Three hours later, all mice were euthanized and peritoneal exudate was collected by flushing the peritoneal cavity with 10 ml of sterile cold 1× PBS and centrifuged at 400×*g* for 10 min at 4°C. Pellet-containing cells were processed for flow cytometric analysis and serum prepared from blood collected by cardiac puncture for cytokine measurement by ELISA (23, 24).

### Flow Cytometry Assay

To quantify the leukocyte recruitment, we performed flow cytometry as described previously (15). Following induction of peritonitis by SP injection, peritoneal lavage was collected and cells were washed with FACS buffer. Peritoneal cells were treated with CD16/32 Fc block (clone93, cat#101320) for 15 min followed by anti-CD45-FITC (clone 30-F11; cat #103108), CD11b-PerCP-Cy5.5 (clone M1/70, cat#101227), and Ly6G-BV421 (clone 1AB, cat#127628) for 45 min to stain neutrophils. Live versus Dead cells were stained using Zombie Yellow™ Fixable viability dye (Biolegend, CA). The data were acquired with BD LSR II flow cytometer (San Jose, CA) and analyzed by FlowJo software version 10.7.2 (Tree Star Inc., Ashland, OR). Neutrophils were gated as CD45^+^CD11b^+^Ly6G^+^ live cells.

### Statistical Analysis

Statistical analyses were performed using GraphPad PRISM software version 9.0.1 (San Diego, CSA). Results were expressed as mean ± standard error of the mean (SEM) values derived from at least three independent experiments. Differences between groups were analyzed by analysis of variance (ANOVA) following by Dunnett’s, Sidak’s and Tukey’s multiple comparisons tests. A p-value less than or equal to 0.05 was considered to be significant.

## Results

### Knockdown of Orai1/2/3 Partially Inhibit SP/MRGPRX2-Mediated Ca^2+^ Influx and Degranulation in LAD2 Cells

**Figure 1:**
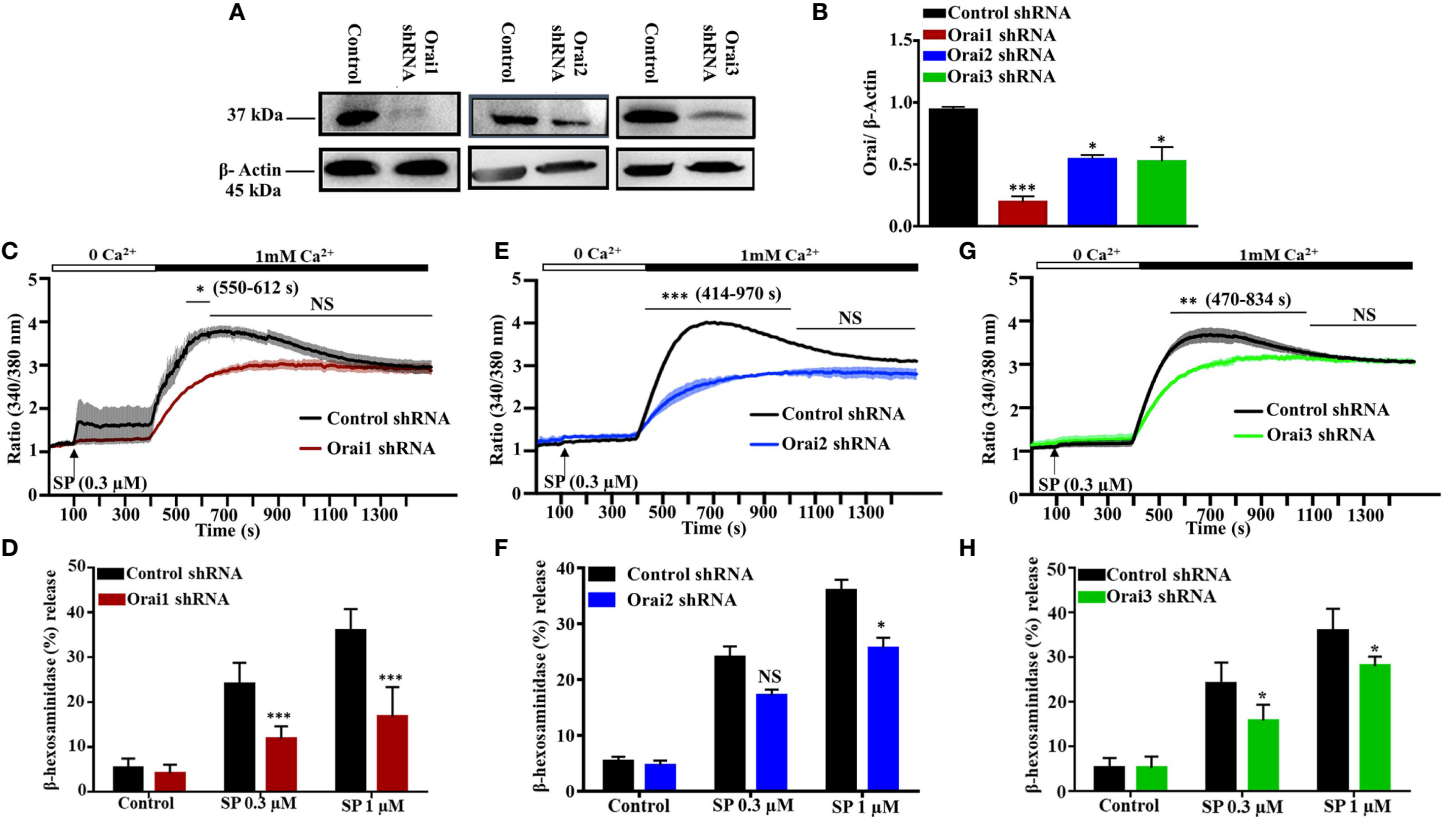
Lentiviral shRNA-Mediated Knockdown of Orai1, Orai2, and Orai3 Reduces SP-Induced Responses in LAD2 Cells. **(A)** Knockdown efficiency of each Orai channel was determined by western blotting in cells transduced with non-targeted (control) or Orai-specific shRNA and a representative blot is shown. **(B)** Quantification of Orai knockdown is represented as bar graph of relative intensities of Orai protein bands normalized to respective β-Actin. **(C, E, G)** Cells were loaded with Fura-2AM, resuspended in Ca^2+^-free buffer (1.5 ml) and measurement of intracellular Ca^2+^ mobilization was initiated *via* the addition of SP (0.3 µM, at 100s). At 400 s, Ca^2+^ concentration of the buffer was increased to 1 mM *via* the addition of 1.5 µl of 1 M calcium and influx was measured for additional 18 min. **(D, F, H)** Effects of Orai knockdown on SP-induced degranulation, as measured by β-hexosaminidase release. Data presented are mean ± SEM of N = 4 experiments. Statistical significance was determined by two-way ANOVA with Tukey’s multiple comparisons at a value ***p < 0.001, **p < 0.01, *p < 0.05, and NS, “not significant”.

All three known Orai isoforms are expressed in human lung MCs and participate in FcϵRI-mediated activation (6). Moreover, in a human MC line, LAD2 cells, shRNA-mediated silencing of STIM-1 results in significant decrease in MRGPRX2-mediated Ca^2+^ mobilization and degranulation (25). Given that STIM-1 couples to Orai1 for Ca^2+^ influx (26), we hypothesized that this CRAC channel contributes to SP-induced responses in human MCs. To test this possibility, we used lentiviral shRNA to individually silence the expression of Orai1, Orai2, and Orai3 in LAD2 cells, which endogenously express MRGPRX2. As shown in **Figures 1A, B**, transduction of LAD2 cells with lentiviral Orai1 resulted in >80% reduction of protein expression when compared to scrambled shRNA control, as determined by western blotting. This was associated with ~50% decrease in SP-induced Ca^2+^ influx (**Figure 1C**) and degranulation (**Figure 1D**). We found that although Orai2 silencing resulted in ~50% reduction in protein expression (**Figures 1A, B** **)**, this was associated with significant inhibition of Ca^2+^ influx (**Figure 1E**) with small reduction in degranulation (**Figure 1F**). Knockdown of Orai3 in LAD2 cells resulted in ~50% decrease in protein expression (**Figures 1A, B**). This was associated with a small but significant reduction in SP-induced Ca^2+^ influx (**Figure 1G**) and degranulation (**Figure 1H**).

### SP-Induced Cytokine/Chemokine Production is Substantially Inhibited in Orai Knockdown LAD2 Cells

**Figure 2:**
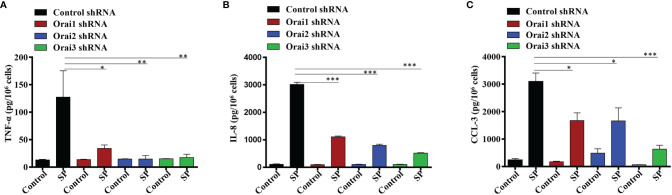
Orai1, Orai2, and Orai3 contribute to SP-Induced Cytokines Generation in LAD2 Cells. LAD2 cells with shRNA-mediated knockdown of each Orai channel or non-targeted (control) cells were exposed to SP (1 µM, for 24 h). Culture supernatants were used to quantitate **(A)** TNF-α, **(B)** IL-8 and **(C)** CCL-3 by ELISA. Data presented are mean ± SEM of N = 3 experiments. Statistical significance was determined by one-way ANOVA with Tukey’s multiple comparisons at a value ***p < 0.001, **p < 0.01 and *p < 0.05.

In addition to degranulation, Orai-mediated Ca^2+^ influx regulates the activities of the transcription factors NFAT and NF-κB, which are required for cytokine/chemokine induction in immune cells (27, 28). We therefore sought to determine the effects Orai1, Orai2 or Orai3 knockdown on SP-induced TNF-α, IL-8, and CCL-3 generation in LAD2 cells. Despite the fact that individual Orai1 and Orai3 knockdown resulted in ~50% inhibition SP-induced Ca^2+^ influx and degranulation **(** **Figure 1**), this resulted in substantial inhibition of SP-induced cytokine/chemokine generation (**Figures 2A–C**). By contrast, knockdown of Orai2 had variable effects on SP-induced TNF-α, IL-8, and CCL-3 generation (**Figures 2A–C**).

### CRAC Channel Inhibitor Synta66 Inhibits SP-Induced Responses in LAD2 Cells

**Figure 3:**
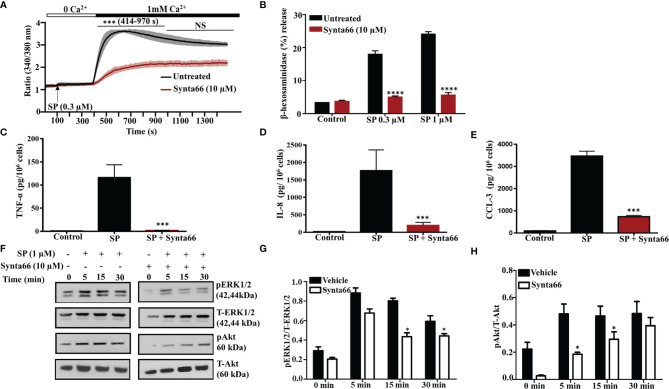
Synta66 Causes Substantial Inhibition of SP-Induced Responses in LAD2 Cells. **(A)** Fura-2AM-loaded LAD2 cells were preincubated with Synta66 (10 µM, 30 min) in Ca^2+^-free buffer and the measurement of intracellular Ca^2+^ mobilization was initiated *via* the addition of SP (0.3 µM) at 100 s. After 400 s, Ca^2+^ concentration of the buffer was increased to 1 mM and Ca^2+^ measurement was continued for additional 18 min **(B)** LAD2 cells were preincubated with Synta66 (10 µM, 5 min), stimulated with the indicated concentrations of SP and β-hexosaminidase release was determined. **(C–E)** Cells were preincubated with Synta66 (10 µM, 30 min) and stimulated with SP (1 µM for 24 h) and culture supernatants were used to measure TNF-α, IL-8 and CCL-3. **(F)** Synta66-preincubated cells were stimulated with SP for different time points and western blotting was performed on cell lysate with anti-pERK1/2, anti-pAkt, total anti-ERK1/2 and total anti-Akt antibodies. **(G, H)** Bar graphs represent relative intensities of bands of phosphoproteins normalized to respective total proteins. Data presented are mean ± SEM of N = 3 experiments. Statistical significance comparing multiple groups was determined by one-way ANOVA with Tukey’s multiple comparisons at a value ****p <0.0001, ***p <0.001, and NS, “not significant” **(A–E)**. Statistical significance was determined by Student’s t-test at a value *p < 0.05 **(G, H)**.

Synta66 is a CRAC inhibitor that inhibits the formation of Orai1:Orai1, Orai1:Orai2, and Orai1:Orai3 dimers (29). Therefore, we used Synta66 to determine the combined role of Orai channels on SP-induced Ca^2+^ influx, degranulation and cytokine/chemokine production. Pretreatment of LAD2 cells with Synta66 (10 µM) resulted in substantial reduction of SP-induced Ca^2+^ influx (**Figure 3A**) but complete inhibition of degranulation (**Figure 3B**), TNF-α, IL-8 and CCL-3 generation **(** **Figures 3C–E** **)**. Involvement of extracellular signal-regulated kinases 1/2 (ERK1/2) and Akt pathways in cytokine production in MCs are well documented (30, 31). Furthermore, MRGPRX2-mediated MC activation leads to increased ERK1/2 and Akt phosphorylation (32, 33). STIM-1 inhibitor SKF pretreatment caused inhibition of ERK1/2 and Akt phosphorylation in LL-37 stimulated MCs *via* MRGPRX2 (25). We therefore sought to determine the effects of Synta66 on ERK1/2 and Akt phosphorylation in LAD2 cells. As shown in **Figures 3F–H**, SP stimulation resulted in time dependent enhanced ERK1/2 and Akt (Ser473) phosphorylation, which were significantly inhibited by Synta66.

### Synta66 Inhibits SP-Induced Ca^2+^ Influx and Degranulation in Primary Human Skin MCs

**Figure 4:**
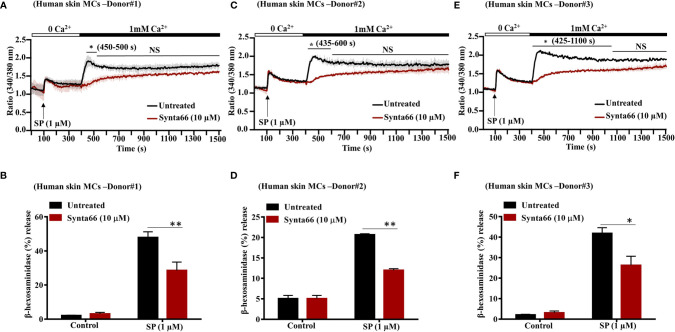
Synta66 inhibits SP-induced Ca^2+^ influx and degranulation in Human Skin Derived MCs. **(A, C, E)** MCs from three donors were pretreated with Synta66 (10 µM, 30 min) and intracellular Ca^2+^ influx was determined in **(B, D, F)**. MCs were preincubated with Synta66, stimulated with SP (1 µM) and β-hexosaminidase release was determined. Data shown are mean ± SEM of three individual experiments from three donors. Statistical significance was determined by two-way ANOVA with Tukey’s multiple comparisons at a value **p < 0.01, *p < 0.05, and NS, “not significant”.

In addition to LAD2 cells, SP causes degranulation in human skin MCs *via* MRGPRX2 (24, 34). To determine the biological relevance of studies with LAD2 cells, we tested the effect of Synta66 on SP-induced Ca^2+^ influx and degranulation in primary human skin-derived MCs isolated from three different donors. Initially, cell surface expression of MRGPRX2 in skin MCs was tested by flow cytometry. Representative flow cytometry traces for MRGPRX2 expression in skin MCs from three healthy donors are shown in **Supplementary Figure 1**. We found that SP triggered Ca^2+^ influx and degranulation in skin-derived MCs in all three donors (**Figures 4A–F**). Stimulation of human skin-derived MCs with SP resulted in Ca^2+^ mobilization from both intracellular stores as well as Ca^2+^ influx from the extracellular medium **(** **Figures 4A, C, E** **)**. Synta66 (10 µM) specifically blocked Ca^2+^ influx with no effect on intracellular Ca^2+^ release **(** **Figures 4A, C, E** **)**. Synta66 also caused significant inhibition in SP-induced degranulation in skin-derived MCs **(** **Figures 4B, D, F** **)**.

### Synta66 Attenuates SP-Induced Degranulation in Mouse PMCs *In Vitro* and Vascular Permeability and Peritonitis *In Vivo*

**Figure 5:**
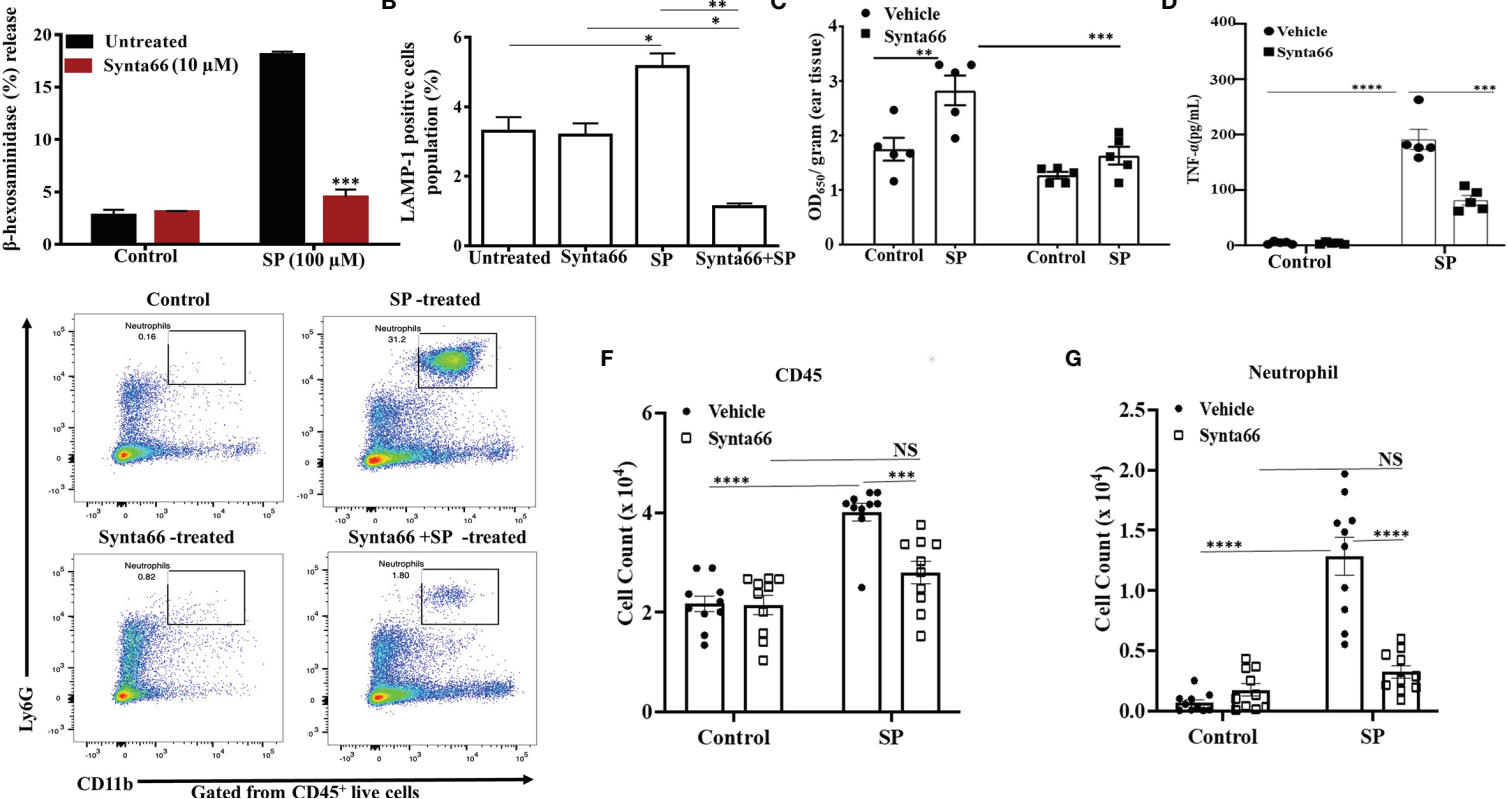
Synta66 Attenuates SP-Induced Murine MC Responses *in vitro* and *in vivo*. SP-induced **(A)** β-hexosaminidase release (N = 4) and **(B)** LAMP-1 expression as determined by flow cytometry on PMCs (N = 3). **(C)** Synta66 or vehicle was administered *via* i.p. injection prior to SP injection in one ear and the buffer control in the other. Vascular permeability was quantified by measuring Evans blue extravasation. Synta66-treated or untreated mice were injected (i.p) with SP. After 3 h, blood and peritoneal lavage fluid was collected (N = 5). **(D)** Serum TNF-α was determined by ELISA, N = 5. **(E)** Representative FACS images showing neutrophil recruitment and numbers indicate the percentage of CD45^+^CD11b^+^ Ly6G^+^ live cells in peritoneal cavity. The absolute number of immune cells was calculated from the flow cytometry profile for each mouse based on gated (50,000 cells) population **(F)** Total CD45^+^ cells, and **(G)** Total neutrophil counts. Data presented are mean ± SEM of N = 10. Statistical significance was determined by two-way ANOVA with Sidak’s multiple comparisons at a value ****p < 0.0001, ***p < 0.001, **p < 0.01, *p < 0.05 and NS, “not significant”.

Our next goal was to determine the effect of Synta66 on MrgprB2-mediated responses in primary mouse PMCs *in vitro* and biological responses *in vivo*. First, we used PMCs from wild-type (WT) and MrgprB2^-/-^ mice to confirm that SP causes degranulation in PMCs *via* MrgprB2. As shown in **Supplementary Figure 2**, SP caused degranulation in WT-PMCs which is completely abrogated in MrgprB2^-/-^ PMCs. Furthermore, we found that Synta66 (10 µM) ablated SP-induced degranulation in mouse PMCs, as measured by both β-hexosaminidase release and cell surface expression of LAMP-1 by flow cytometry (**Figures 5A, B**). Gating strategy for LAMP-1 expression by flow cytometry was shown in **Supplementary Figure 3**. To determine the effect of blocking Orai channels on mouse cutaneous MCs degranulation *in vivo*, we administered Synta66 or vehicle intraperitoneally (i.p) followed by Evans blue dye injection through intravenous route. SP was then injected into one ear and vehicle into the other. The data presented in **Figure 5C** clearly demonstrate that, consistent with its effect on human skin MCs (**Figure 4**), Synta66 inhibited murine cutaneous MC degranulation *in vivo* as determined by a significant reduction in SP-induced Evans blue dye extravasation.

We also sought to determine if Synta66 inhibits cytokine production and the recruitment of inflammatory cells *in vivo*. Mice were injected (i.p) with Synta66 prior to intraperitoneal injection with SP or vehicle. Three hours later, serum TNF-α concentration and neutrophil recruitment in peritoneal lavage (CD45^+^CD11b^+^Ly6G^+^ live cells) were quantitated. As shown in **Figure 5D**, serum TNF-α level was significantly reduced in Synta66-treated mice. SP injection caused recruitment of inflammatory cells at peritoneal cavity and Synta66 substantially inhibited SP-induced leukocyte recruitment especially neutrophils **(** **Figures 5E–G** and **Supplementary Figure 4** **).**

## Discussion

Ca^2+^ is an indispensable second messenger signaling for agonist induced MC activation and mediator release (35). FcϵRI and MRGPRX2-mediated signaling utilize both shared and distinct pathways that lead to Ca^2+^ mobilization and degranulation. Both FcϵRI and MRGPRX2 mediated signaling caused comparable level of Ca^2+^ mobilization, however there is marked differences in spatiotemporal pattern of granule exocytosis and mediator content (16). Moreover, FcϵRI stimulation cause PLCγ dependent Ca^2+^ influx while MRGPRX2 couples to Gαq to promote Ca^2+^ influx (35, 36). Identification of Ca^2+^ channel in FcϵRI-mediated signaling is well characterized and Orai plays a crucial role in antigen-IgE-dependent MC activation (6–8, 37, 38). However, the possibility that MC activation *via* MRGPRX2/MrgprB2 requires Orai channels has not been determined. Using shRNA-mediated knockdown of Orai1/Orai2/Orai3 and a pharmacological inhibitor, we provide the first demonstration that Orai channels are involved in MRGPRX2/MrgprB2-mediated MC activation *in vitro* and vascular permeability and leukocytes recruitment *in vivo*.

Ashmole et al. demonstrated that transcripts of all three known Orai channels are expressed in human lung MCs. Moreover, Orai1 mRNA is most abundant isoform in human lung MCs (6). However, our data showed comparable expression of all three Orai proteins in LAD2 cells which suggests there might be variable level expression of Orai isoforms in different MC lineage. Knockdown of Orai1 resulted in inhibition of FcϵRI-dependent Ca^2+^ influx and mediator release (7). Similarly, we found that Orai1 significantly contributes to MRGPRX2-mediated Ca^2+^ influx and mediator release in LAD2 cells. Tsvilovskyy et al., recently showed that absence of Orai2 in mouse PMCs results in enhanced FcεRI and MrgprB2-mediated Ca^2+^ mobilization and degranulation when compared to control cells (38). Based on this finding, it was proposed that Orai2 serves as a negative regulator for MCs degranulation *via* two possible mechanisms. One involving the formation of a heterodimer with Orai1 and thus preventing its activation and the other resulting in the formation of Orai2-STIM-1 complex rendering insufficient STIM-1 available for Orai1 activation. However, shRNA-mediated downregulation of Orai2 in human lung MCs has marginal inhibitory effects on IgE-mediated Ca^2+^ mobilization and degranulation (7). Our data demonstrated that although Orai2 knockdown caused significant reduction in Ca^2+^ mobilization, it has minimum effect on degranulation. The reason for the difference is not clear but could reflect differences in the expression level of Orai1 and Orai2 in human and rodent MCs. Thus, while human lung MCs and LAD2 cells express Orai1 at >10-fold higher than that of Orai2, rodent MCs express both proteins at similar levels (8, 39). In addition, unlike Orai1, majority of the Orai2 is found in intracellular sites in rat basophilic leukemia (RBL-2H3) cells and the possibility that intracellular Orai2 could modulate Ca^2+^ influx has been suggested but the mechanism is not clear (39). Ashmole et al., successfully utilized adenoviral shRNA to silence the expression of Orai1 and Orai2 but not Orai3 in human lung MCs (7). However, our attempt to knockdown Orai3 in LAD2 cells using lentiviral shRNA was more successful and resulted in partial reduction in SP-induced Ca^2+^ influx and degranulation.

Given that Orai1 and Orai3 are expressed at similar levels in human primary MCs and LAD2 cells, it is possible that Orai1:Orai3 dimer contributes to SP-induced signaling and mediator release (8). Because we were unable to silence the expression of Orai3 to >50% in LAD2 cells, it was not feasible to simultaneously reduce the expression both Orai1 and Orai3. Synta66 is an established pharmacological inhibitor that blocks homo- and heterodimer formation of Orai channel (29). Furthermore, Synta66 causes substantial inhibition of IgE-mediated degranulation in human lung MCs and LAD2 cells and blocks allergen-induced bronchial smooth muscle contraction *ex vivo* (6, 7). However, because Orai2 is expressed at low level in MCs and found predominantly in the cytoplasm, any inhibitory effect of Synta66 likely reflects its ability to target Orai1 and Orai3 (8, 39). Here, we also found that Synta66 caused complete inhibition of SP-induced Ca^2+^ influx and degranulation, indicating probable involvement of Orai1:Orai3 dimer in MRGPRX2-mediated MC activation. Along with cultured MCs, Synta66 exhibited similar inhibitory effect on primary human skin-derived MCs indicating its biological relevance.

Calcium influx initiates a cascade of downstream signaling that lead to calmodulin–calcineurin dependent NFAT1 and NF-κβ activation resulted in transcription of cytokine genes (40, 41). Calcium-dependent NFAT activation caused IL-17 and TNF-α secretion associated with autoimmune disease rheumatoid arthritis (42). Orai1 also modulates Th1 and Th17 responses in experimental autoimmune encephalomyelitis (43). Moreover, Orai also contributes FcϵRI-mediated release of an array of inflammatory cytokines such as TNF-α, IL-5, IL-6, IL-8, and IL-13 from human lung MCs (8). The data presented herein demonstrate that knockdown of individual Orai channel significantly reduce proinflammatory cytokine/chemokine secretion. Blocking of Orai channel using pharmacological inhibitor showed complete ablation of cytokine/chemokine generation which may be due to its inhibitory effect on multiple Orai isoforms. In agreement with the cytokine production Synta66 pretreatment caused inhibition of ERK1/2 and Akt phosphorylation.

As for human MCs, rodent MCs also express all three Orai family members (44). Orai1 plays a predominant role in Ca^2+^ signaling in murine T-cells *in vitro* and *in vivo*. Nonfunctional Orai1 knock-in mice showed impaired Ca^2+^ influx *in vitro* and reduced delayed hypersensitivity and colitis *in vivo* (45). Although mutation in Orai1 in human patient has no major phenotypic effect, Orai1^−/−^ mice cannot survive (46–48). Therefore, use of pharmacological inhibitor for Orai could overcome the lethality associated with genetic deletion of Orai1 *in vivo*. Our data on isolated peritoneal MCs demonstrated complete inhibition of SP-induced degranulation by Synta66. MC degranulation is associated with enhanced vascular permeability and cutaneous anaphylaxis. Synta66 pretreatment inhibited SP-induced vascular permeability (early MC response) and reduced immune cell recruitment and cytokine production (delayed MC response).

In conclusion, we have utilized gene-silencing and pharmacological strategies to show that multiple Orai/CRAC channels contribute to SP-induced MRGPRX2-mediated Ca^2+^ influx, degranulation, TNF-α, IL-8 and CCL-3 generation in MCs *in vitro*. Most importantly, we found that SP-induced increased vascular permeability and neutrophil recruitment, which are mediated *via* MrgprB2, are blocked by Synta66. These findings suggest that Orai channel inhibitors could serve as a novel strategy for the modulation of neurogenic inflammation, pain, chronic urticaria, mastocytosis and atopic dermatitis.

## Data Availability Statement

The original contributions presented in the study are included in the article/**Supplementary Material**. Further inquiries can be directed to the corresponding author.

## Ethics Statement

The studies involving human participants were reviewed and approved by the Internal Review Board at the University of South Carolina. The patients/participants provided their written informed consent to participate in this study. The animal study was reviewed and approved by the Institutional Animal Care and Use Committee at University of Pennsylvania.

## Author Contributions

HA contributed to conception, supervision and funding acquisition of the study. SC, IA, SR, and AA performed experiments and analyzed the data. SC, SR, and HA wrote the first draft of the manuscript. YH and CO provided human skin MCs for the study. All authors contributed to the article and approved the submitted version.

## Funding

This work was supported by the National Institutes of Health grants R01-AI124182, R01-AI143185 and R01-AI149487 to HA, and R21-AR067996 and P20 GM-103641 to CO.

## Conflict of Interest

The authors declare that the research was conducted in the absence of any commercial or financial relationships that could be construed as a potential conflict of interest.

## Publisher’s Note

All claims expressed in this article are solely those of the authors and do not necessarily represent those of their affiliated organizations, or those of the publisher, the editors and the reviewers. Any product that may be evaluated in this article, or claim that may be made by its manufacturer, is not guaranteed or endorsed by the publisher.

## Supplementary Material

Supplementary Figure 1 Cell Surface Expression of MRGPRX2 in Human Skin MCs from Three Donors. Skin MCs were incubated with PE-conjugated anti-MRGPRX2 antibody (blue histograms) or isotype control antibody (grey histograms) and receptor expression was assessed by flow cytometry N=3.

Supplementary Figure 2 SP Induces Degranulation in Mouse PMCs *via* MrgprB2. Peritoneal MCs from WT and MrgprB2^-^/^-^ mice were exposed to SP and β-hexosaminidase release was determined. Data presented are mean ± SEM of N=3 experiments. Statistical significance was determined by two-way ANOVA with Tukey’s multiple comparisons at a value ***p < 0.001.

Supplementary Figure 3 Flow Cytometry Gating Strategy of LAMP1 Expression in PMCs N=3.

Supplementary Figure 4 Flow cytometry gating strategy of peritoneal lavage immune cells. Numbers indicate the percentage of cells pre-gated on viability. Debris- cells, single cells, CD45^+^ live cells, CD45^+^CD11b^+^Ly6G^+^ live cells (neutrophils).

## Abbreviations

CRAC, calcium release-activated calcium; FITC, fluorescein isothiocyanate; GPCR, G Protein-coupled receptor; IL-8, interleukin-8; TNF-α, Tumor Necrosis Factor-alpha; LAMP-1, lysosomal-associated membrane protein-1; MCs, mast cells; MFI, mean fluorescent intensity; MrgprB2, Mas-related G protein-coupled receptor B2; MRGPRX2, Mas-related G protein-coupled receptor X2; PE, phycoerythrin; PMCs, peritoneal mast cells; STIM-1, stromal interaction molecule-1; SOC, store-operated calcium; SP, substance P; WT, wild-type.
