# Supplementary figures and images for: Inhibition of Orai Channel Function Regulates Mas-Related G Protein-Coupled Receptor-Mediated Responses in Mast Cells
Source: Front Immunol. 2022 Jan 20;12:803335. doi: 10.3389/fimmu.2021.803335 (PMC8810828; doi:10.3389/fimmu.2021.803335)

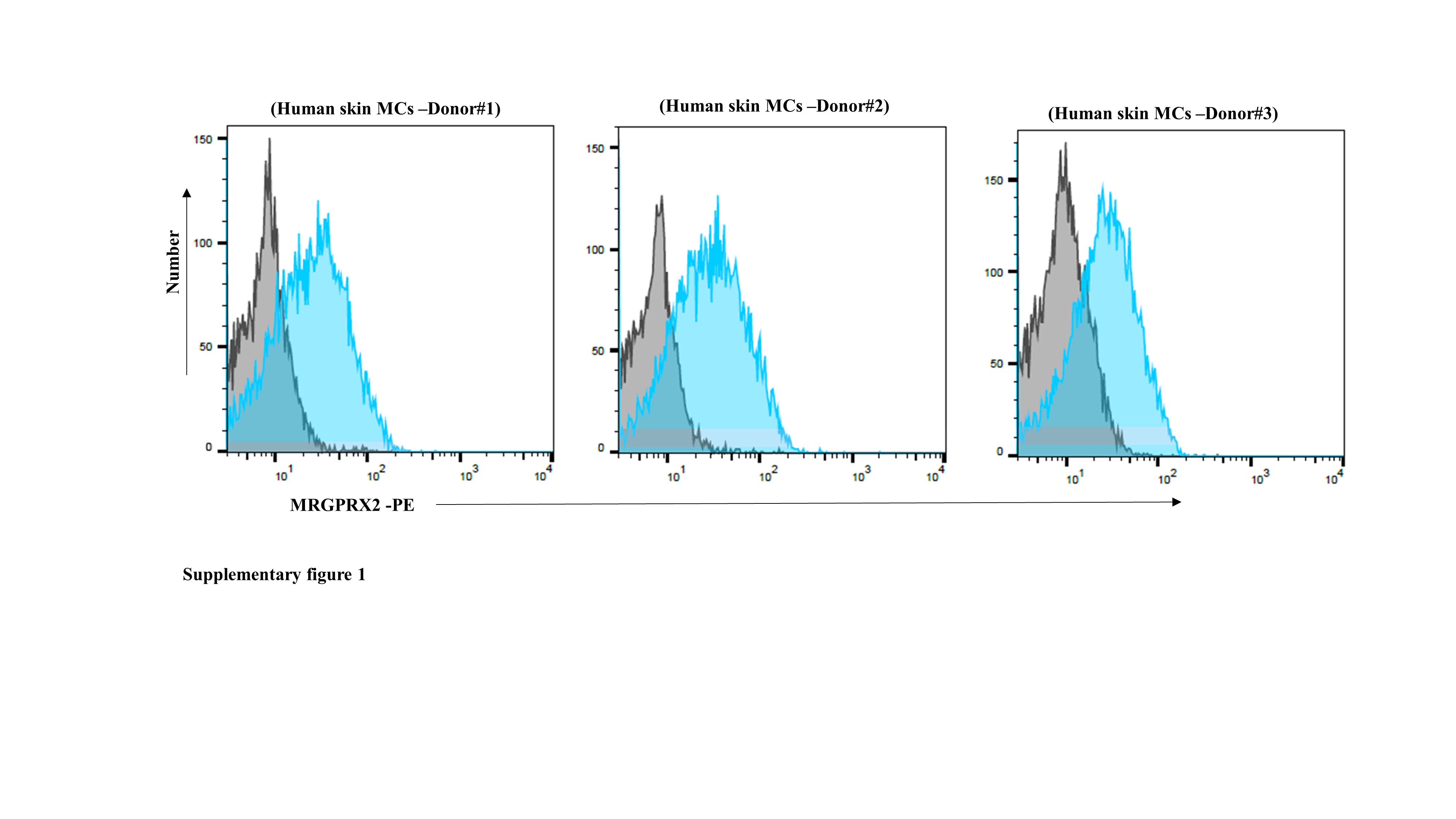

Supplement: Supplementary Figure 1 — Cell Surface Expression of MRGPRX2 in Human Skin MCs from Three Donors. Skin MCs were incubated with PE-conjugated anti-MRGPRX2 antibody (blue histograms) or isotype control antibody (grey histograms) and receptor expression was assessed by flow cytometry N=3. [file Image_1.jpeg]

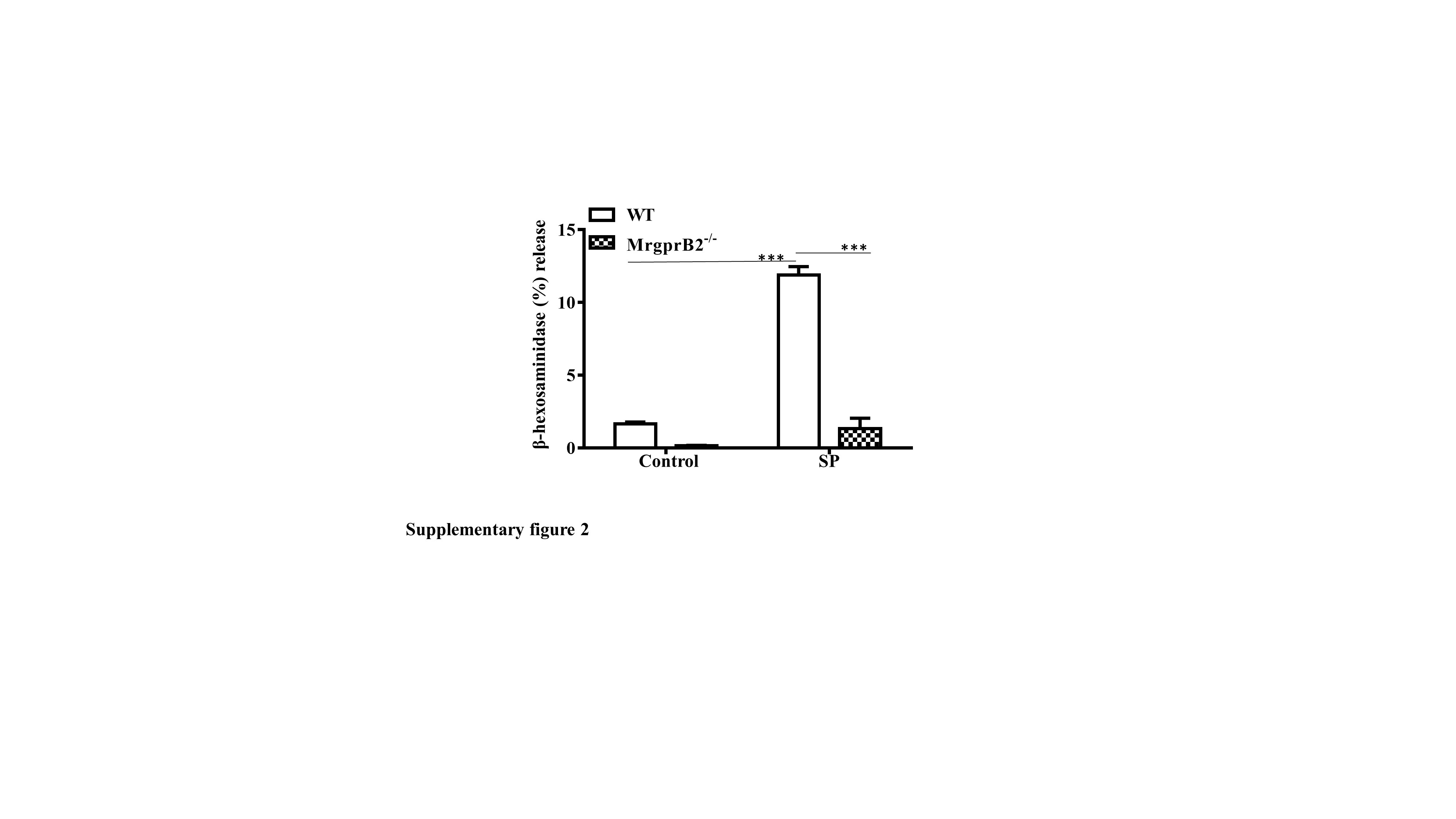

Supplement: Supplementary Figure 2 — SP Induces Degranulation in Mouse PMCs via MrgprB2. Peritoneal MCs from WT and MrgprB2-/- mice were exposed to SP and β-hexosaminidase release was determined. Data presented are mean ± SEM of N=3 experiments. Statistical significance was determined by two-way ANOVA with Tukey’s multiple comparisons at a value ***p < 0.001. [file Image_2.jpeg]

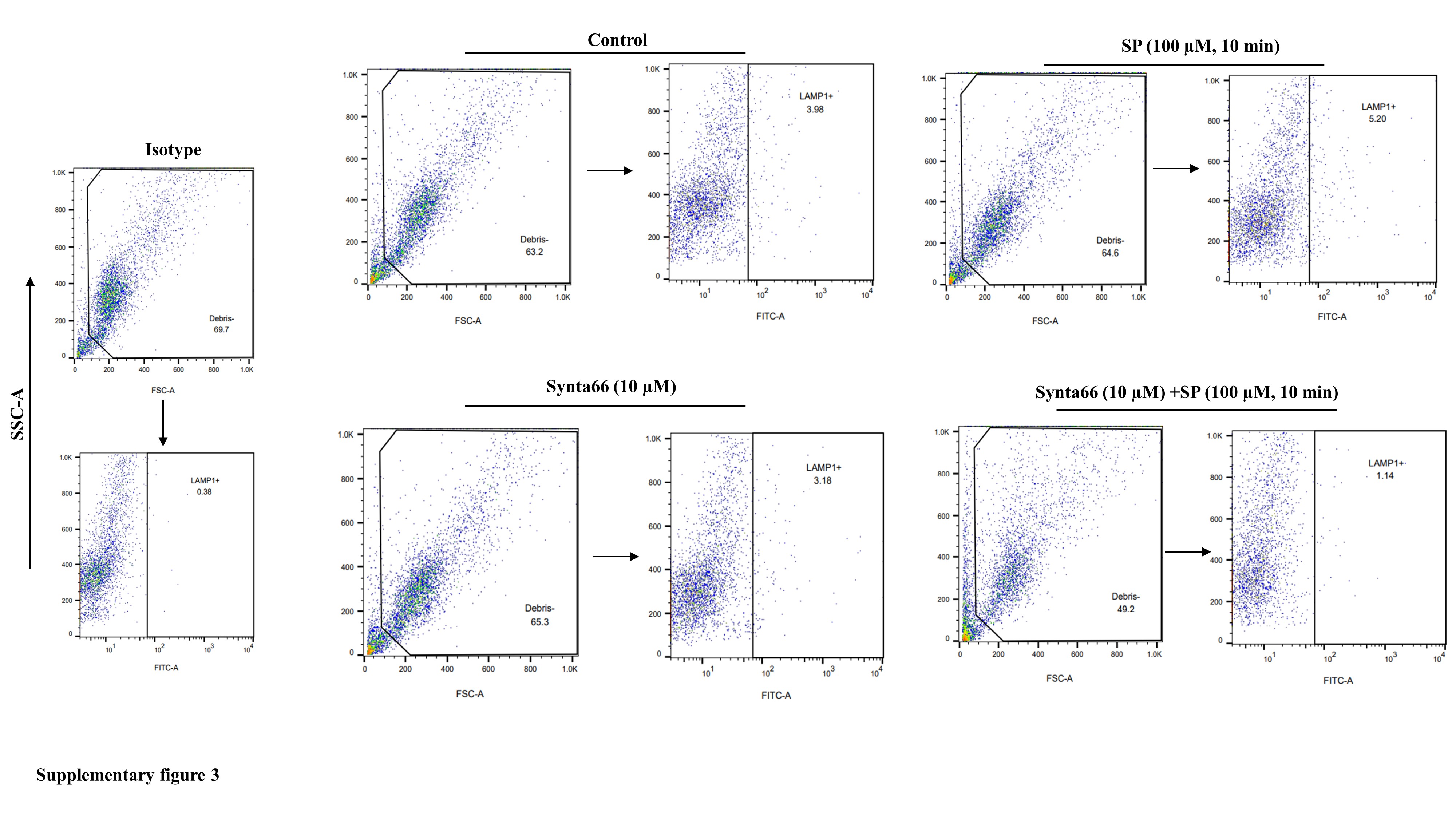

Supplement: Supplementary Figure 3 — Flow Cytometry Gating Strategy of LAMP1 Expression in PMCs N=3. [file Image_3.jpeg]

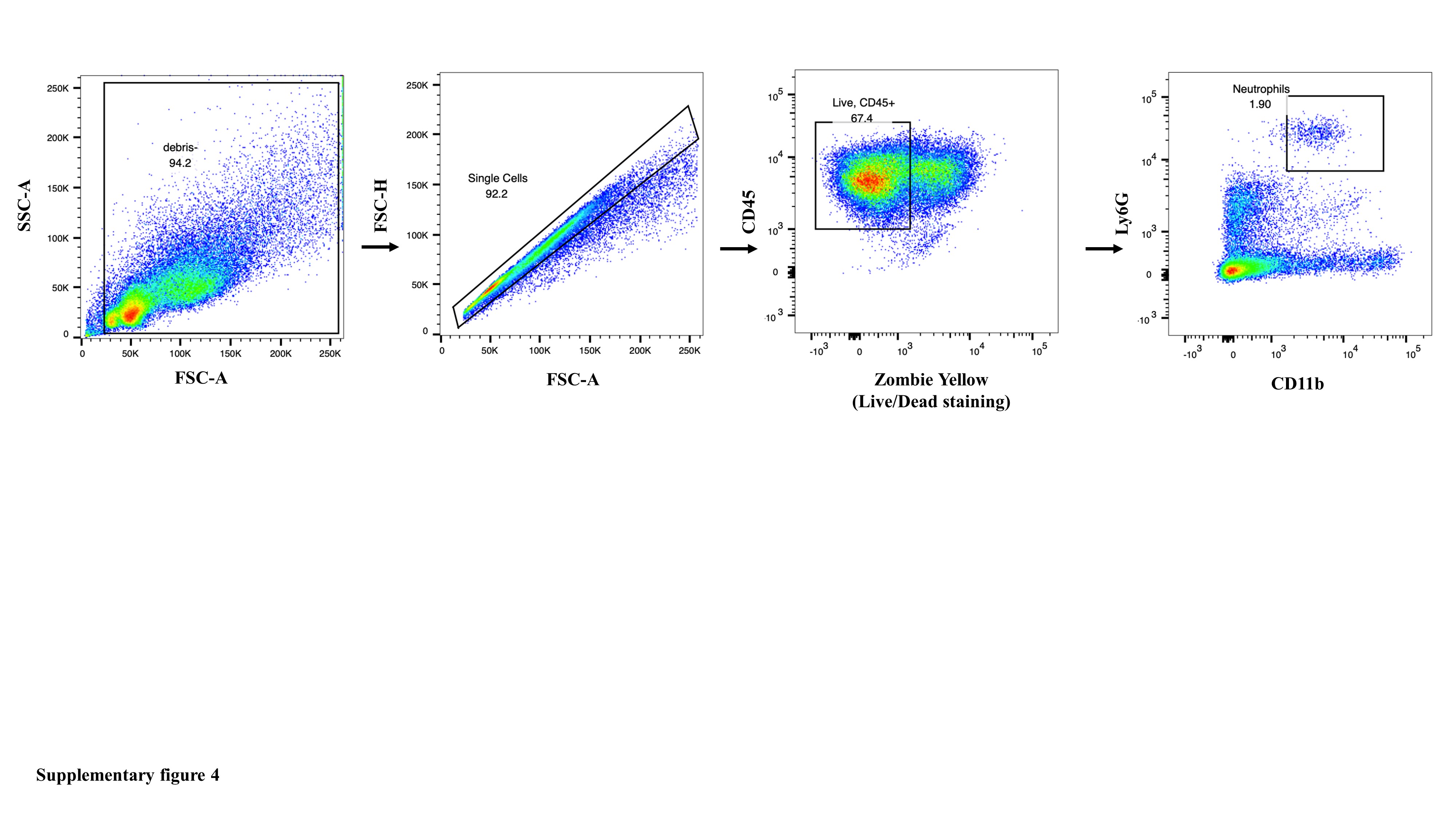

Supplement: Supplementary Figure 4 — Flow cytometry gating strategy of peritoneal lavage immune cells. Numbers indicate the percentage of cells pre-gated on viability. Debris- cells, single cells, CD45+ live cells, CD45+CD11b+Ly6G+ live cells (neutrophils). [file Image_4.jpeg]
